# Thiazine-2-thiones as Masked 1-Azadienes in Cascade Dimerization Reactions

**DOI:** 10.3390/molecules22040541

**Published:** 2017-03-28

**Authors:** Art Kruithof, Christophe M. L. Vande Velde, Eelco Ruijter, Romano V. A. Orru

**Affiliations:** 1 Department of Chemistry & Pharmaceutical Sciences and Amsterdam Institute for Molecules, Medicines & Systems, Vrije Universiteit Amsterdam, De Boelelaan 1108, 1081HZ Amsterdam, The Netherlands; a.kruithof@vu.nl (A.K.); e.ruijter@vu.nl (E.R.); 2 Faculty of Applied Engineering, Advanced Reactor Technology, University of Antwerp, Groenenborgerlaan 171, 2018 Antwerpen, Belgium; christophe.vandevelde@uantwerpen.be

**Keywords:** 1-azadienes, multicomponent reaction, thiazine-2-thiones, tetrahydropyrimidines, *aza*-Diels-Alder, chemical space, molecular diversity, microwave

## Abstract

We report the unexpected formation of a 1-azadiene dimer from 4,6-diphenyl-3,6-dihydro-2*H*-1,3-thiazine-2-thiones under prolonged microwave irradiation. In this manner, thiazine-2-thiones act as “masked” 1-azadiene equivalents, which makes them useful synthetic tools to access complex heterocyclic frameworks. We compare this dimerization with earlier approaches and elaborate on the observed diastereoselectivity.

## 1. Introduction

To find truly novel bioactive scaffolds it is important to cover chemical space effectively [1] and innovative synthetic methodology is highly desired. In the past, we showed that multicomponent reactions (MCRs) are useful synthesis platforms in this respect [2,3,4,5]. A thus far scarcely employed strategy is the in-situ derivatization of known MCR products via dimerization cascades. This would allow to cover chemical space [6,7,8,9,10,11] towards regions that were less explored thus far.

**Scheme 1:**
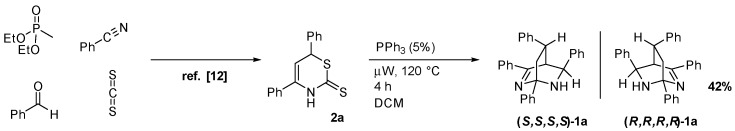
Synthesis of **1a** by microwave irradiation of thiazine **2a**.

We previously reported the reaction of in situ formed 1-azadienes with carbon disulfide towards 3,6-dihydro-2*H*-1,3-thiazine-2-thiones [12]. Relevant for the current paper is that we found a rather unexpected diastereoselective transformation of 4,6-diphenyl-3,6-dihydro-2*H*-1,3-thiazine-2-thione (**2a**) to bridged bicyclic aminals **1a** under microwave irradiation at 120 °C in dichloromethane (Scheme 1). Here we elaborate on this unusual synthesis of bicyclic tetrahydropyrimidines [13,14].

**Scheme 2:**
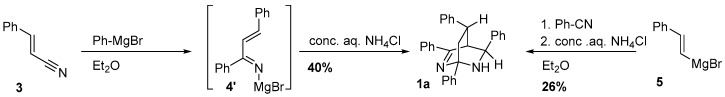
Synthesis of **1a** by Wright et al. [15].

It should be noted that, although bicyclic tetrahydropyrimidines can be found throughout the literature, a structure-based search returned only a handful of examples of the specific bicyclic heterocyclic core of **1a**. Already in 1950 Wright et al. (Scheme 2) [15] reported a reaction between cinnamonitrile (**3**) and phenylmagnesium bromide to give **1a** in 40% yield. Based on the reported melting point, this reaction probably provides the same mixture of all-*S*/all-*R* stereoisomers. The inverse reaction, e.g., the reaction between benzonitrile and styrylmagnesium bromide (**5**), also proved successful, albeit in lower yield.

In both cases dimerization was reported to specifically take place by quenching the organomagnesium species with a cold concentrated aqueous ammonium chloride solution. In line with chemical intuition, quenching with other acidic aqueous solutions gives the hydrolysis product, i.e., the corresponding chalcone [16]. Praiseworthily, the authors propose the proper structure based solely on elemental analysis and degradation products. Understandably, there is no mention about the relative stereochemistry.

**Scheme 3:**
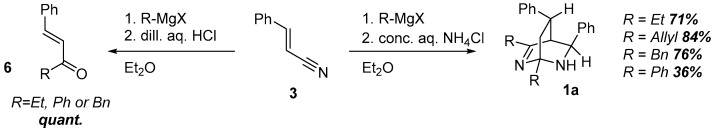
Synthesis of 1a by Henze et al. [17].

Shortly after the first report, Henze et al. communicated the synthesis of three more examples in good yields (Scheme 3) [17]. The observed increased yields for alkylmagnesium bromides in comparison to phenylmagnesium bromide suggests that increased electron density on the terminal imine is beneficial for this reaction. Furthermore, the authors again showed that the intermediate Grignard adduct of phenylmagnesium bromide and cinnamonitrile only reacts to form the heterocyclic dimer using aqueous ammonium chloride solution, as quenching of the Grignard adduct with diluted aqueous hydrochloric acid only gave the corresponding chalcones **6**.

Conveniently, Forrester et al. used the procedure by Wright et al. in a more recent study towards the preparation of triarylpyridines and -pyrimidines also reporting the corresponding ^1^H and ^13^C-NMR data [18]. This conclusively confirms the diastereoisomer formed via our dimerization cascade reaction of 4,6-diphenyl-3,6-dihydro-2*H*-1,3-thiazine-2-thione (**2**) is the same diastereoisomer as obtained via the addition of phenylmagnesium bromide to cinnamonitrile. Finally, it should be noted that Nitta et al. reported **1a** as one of the products in their studies of a Mo(CO)_6_ induced reductive cleavage of oximes in ethanol and an acetonitrile-water mixture [19].

## 2. Results and Discussion

**Figure 1:**
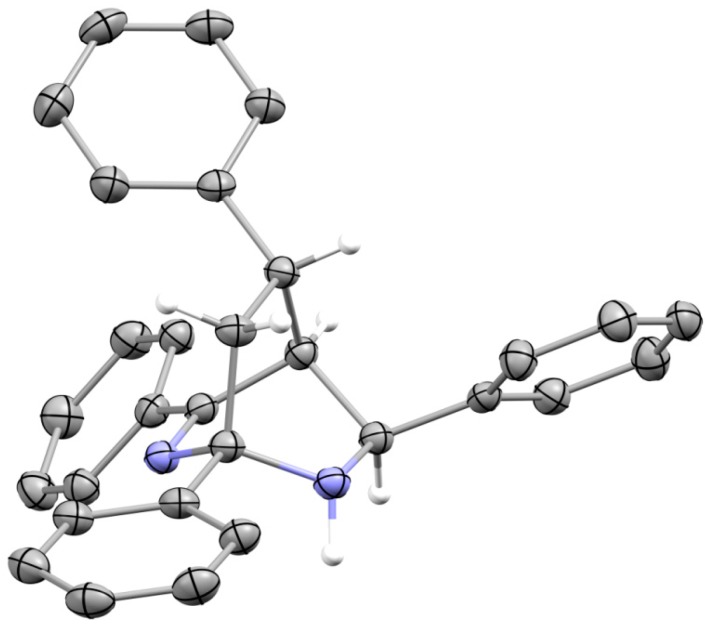
X-ray structure of **1a**, depicted as the *S,S,S,S*-enantiomer; aromatic hydrogen atoms omitted for clarity.

Thus, when we treated thiazine thione **2a** under microwave irradiation at 120 °C, we observed the diastereoselective formation of bicyclic tetrahydropyrimidines **1a** (Scheme 1). TLC analysis showed full conversion after 4 h and serendipitously it was found that the addition of 5% of triphenylphosphine leads to quite a clean conversion to the bicyclic heterocyclic products. In this way, dimer **1a** was isolated in 42% yield after column chromatography. Although 2D-NMR experiments already established the exact structure of **1a**, X-ray crystallographic analysis conclusively confirmed the diastereoselective formation of the all-*S*/all-*R* racemic mixture (Figure 1). All NMR spectra can be found in the Supplementary Materials to which a link can be found at the end of this article.

**Scheme 4:**
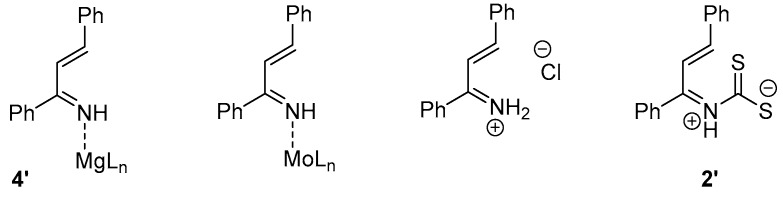
Possible activation modes of the different reports on 1-azadiene dimerization.

As the reaction conditions described in the introduction for the dimerization process to generate bicyclic pyrimidines like **1** differ considerably, we assume that two “liberated” 1-azadiene fragments are condensed. Either Lewis or Brønsted acid activation by magnesium ions, molybdenum species or by ammonium chloride may be important. In our dimerization cascade a covalent activation by carbon disulfide (**2’**), resulting from the ring opening of **2** may even occur (Scheme 4).

**Scheme 5:**
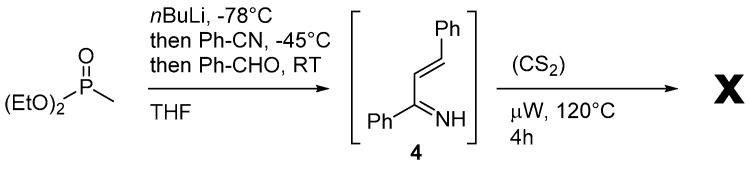
Horner-Wadsworth-Emmons (HWE) reaction towards 1-azadiene **4b** [12] and subsequent attempts towards dimerization.

Furthermore, we note that attempts to dimerize in situ formed 1-azadiene **4** (via our well-known Horner-Wadsworth-Emmons (HWE) approach) in the presence or absence of carbon disulfide proved not successful. A complex mixture of reaction products was formed and no bicyclic heterocyclic products of type **1a** could be detected (Scheme 5).

Relating this to the reaction outcome as depicted in Scheme 1 it suggests that at 120 °C the thiazine-2-thione (**2a**) slowly undergoes a formal *retro*-Diels-Alder reaction to liberate 1-azadiene **4**, reducing the number of side reactions. Alternatively, **2a** could undergo ring opening to produce the 1-azadiene CS_2_ adduct (**2’**; Scheme 4). 

It should be noted that the basic conditions ((EtO)_2_POOLi, slight excess of *n*BuLi) of the HWE reaction leading to **4** may hamper subsequent dimerization. Thus, in the dimerization route via thiazine-2-thiones the latter seem to act as “masked” 1-azadiene equivalents yielding the bicylic products **1a** in a much cleaner fashion.

The above findings in combination with a detailed retrosynthetic analysis taking into account the observed diastereoselectivity led to a plausible reaction mechanism for this cascade dimerization process [20].

**Scheme 6:**
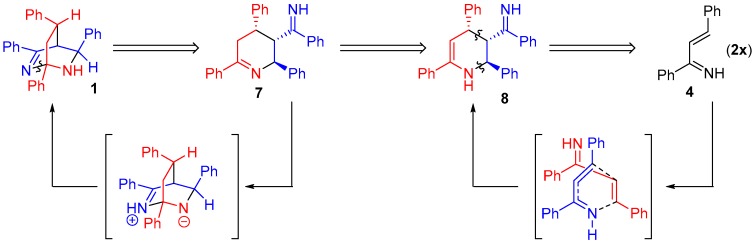
Retrosynthetic analysis and concerted *aza*-Diels-Alder mechanism.

In total, three new σ-bonds are formed in this reaction. The most labile or polarized is the N–C bond of the aminal functionality which is destabilized by the adjacent π-bond (Scheme 6). This would indeed be the first bond for a retrosynthetic disconnection.The remaining six-membered ring **7** is the (formal) hetero-Diels-Alder adduct of two azadiene molecules.

A first obvious mechanistic option for cyclization would be an *aza*-Diels-Alder reaction/tautomerization process. Reasoning from the connectivity in **8**, the orientation of the diene and dienophile as depicted in Scheme 6 gives the best HOMO-LUMO overlap. Secondly, the observed diastereoselectivity implies that the conformation of the dienophile follows the *endo* rule, giving the most stabilized transition state by beneficial π-π interactions. Again, covalent bonding or Brønsted/Lewis acid interactions, analogous to the structures shown in Scheme 4, may promote these reactions.

A concerted *aza*-Diels-Alder mechanism nicely accounts for the diastereoselectivity observed, therefore it seems a likely pathway under the aprotic conditions of our reaction. However, under the protic reaction conditions reported earlier by others (see introduction), clearly a more step-wise mechanistic pathway involving (zwitter)ionic species that could tautomerize instantly cannot be excluded.

**Scheme 7:**
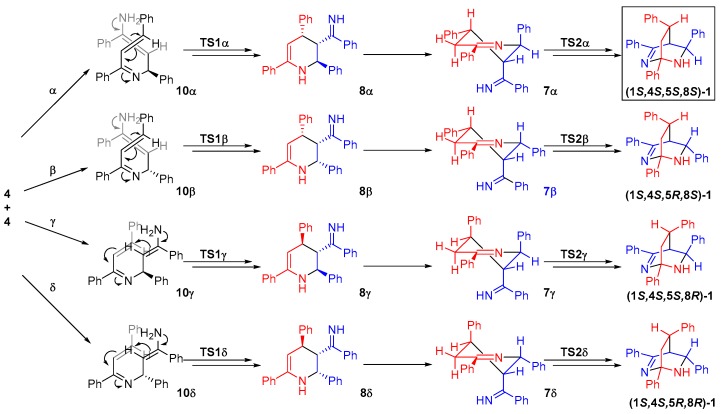
Possible stepwise mechanism.

Rationalization of all four possible ring closing pathways shows that only pathway **α** leads to the observed diastereoisomer (Scheme 7). The four pathways are distinctly different in terms of *endo*/*exo* and *syn*/*anti* in relation to the chiral center already present. Therefore, especially at 0 °C, a stepwise mechanism could indeed lead to selective product formation as well.

The 1-azadiene partner that acts as the electrophile can adopt two possible conformations, i.e., *s-cis* and *s-trans*, and thus both the *E* and *Z*-isomer of enamine **10** can formally exist. However, for simplicity both forms are treated the same, in view of the fact that in cyclic intermediates **7** and **8** rotation around the former C3–C4 bond can occur and the orientation in the final product is fixed.

In the first cyclization, the exocyclic position of the enamine moiety in **TS1γ** and **TS1δ** would be sterically favored over the endocyclic position in **TS1α** and **TS1β**. On the other hand, a π-π interaction, analogous to the *endo*-rule, favors the *endo*-attack over the *exo*-attack. Additionally, in the enamine **8γ** the substituents can all adopt pseudoequatorial positions, whereas **8α**, **8β** and **8δ** have at least one of the three substituents in pseudoaxial position in all possible conformations. If the system complies with the Bell-Evans-Polanyi principle [21,22], this would mean **TS1γ** is likely to be the lowest in energy.

Because the ring closure towards **8** is very likely irreversible, selectivity in **TS2** will not change the outcome of the reaction. However, for clarity and completeness: after the tautomerization towards the tetrahydropyridines **7δ**, the exocyclic imine needs to adopt a pseudoaxial position in order for the reaction to proceed. This entails that **TS2γ** would be least favored with both phenyl rings in a pseudoaxial arrangement, followed by **TS2α** and **TS2δ** with one pseudoaxial and one pseudoequatorial substituent. **TS2β** is the most favored as both phenyl rings are pseudoequatorial.

For all reported dimerizations towards **1**, a formal hetero-Diels-Alder-type mechanism explaining the observed diastereoselectivity is most likely, although a stepwise mechanism cannot be excluded under protic reaction conditions. There is, however, no evident indication that the diastereoselectivity in the first step of the stepwise mechanism would favor formation of the observed diastereoisomer. 

**Scheme 8:**
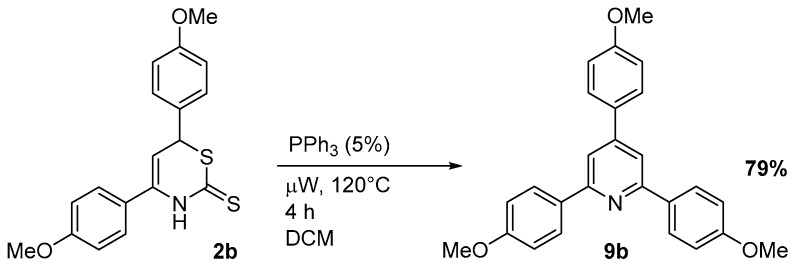
Formation of **9b** by microwave irradiation of thiazine **2b**.

We decided to investigate whether an increase in electron density on the terminal imine would, analogously to the studies by Henze et al. [17], result in higher yields of our dimerization cascade. Thus, bismethoxy-thiazine-2-thione **2b** (obtained from our HWE-based MCR) was reacted under the optimized reaction conditions (Scheme 8). However, instead of the expected bicyclic aminal, pyridine **9b** was isolated in 79% yield.

**Scheme 9:**
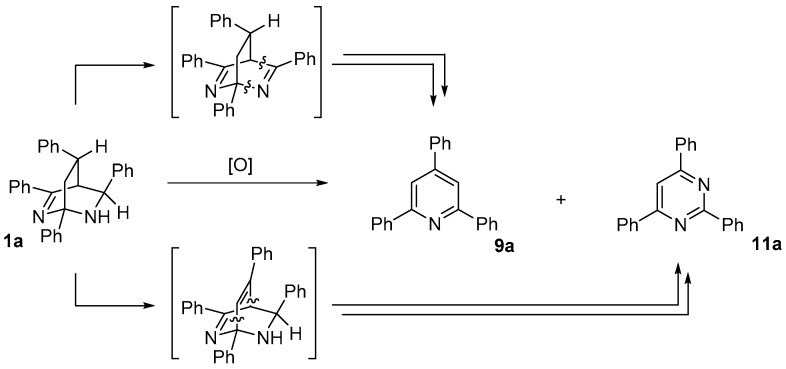
Oxidation of **1a** to pyridine **9a** and pyrimidine **11a** reported by Forrester et al. [18].

This result is in line with the aforementioned report by Forrester et al. in which **1a** is oxidized with potassium persulfate to triphenylpyridine **9a** and triphenylpyrimidine **11a** (Scheme 9) [18]. Alternatively, with di-*tert*-butyl peroxide only pyrimidine **11a** was isolated. In this context it is postulated that this fragmentation likely proceeds via a formal *retro*-Diels-Alder pathway after an initial oxidation of either the N–C bond or the C–C bond, followed by a second oxidation, leading to the pyridine or the pyrimidine, respectively. In principle, the electron donating *para*-methoxy groups provide more electron density to the ringsystem and thus oxidation is less cumbersome. As a result, the bicyclic intermediate may immediately undergo the formal *retro*-Diels-Alder reaction towards pyridine **9b**. Additionally, without a dedicated oxidation agent present, the N–C bond would be more prone to oxidation, giving selectively the pyridine.

**Scheme 10:**
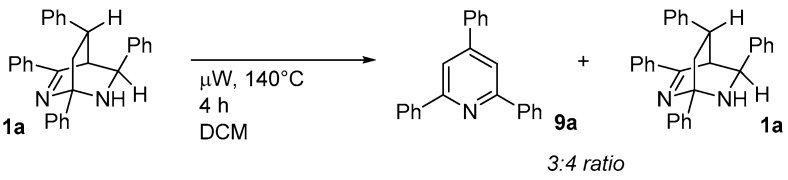
Fragmentation of **1a** to triphenylpyridine **9a**.

In order to show that the pyridines **9** can be formed via bridged aminals **1** under the non-oxidative conditions of the reaction, we heated **1a** in dichloromethane at 140 °C under microwave irradiation for 4 h (Scheme 10).

^1^H-NMR analysis of the crude product clearly shows, along with some minor side products, the distinct peaks of triphenyl pyridine **9a**, in a 3:4 ratio with the starting material **1a**. This demonstrates that bridged aminals **1** are indeed able to fragment and give pyridines **9** without the addition of an oxidant, and thus that pyridine **9b** is likely formed via this bicyclic intermediate.

**Scheme 11:**
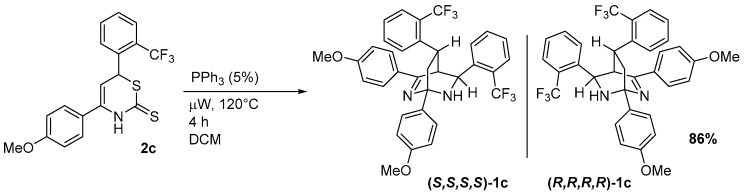
Synthesis of **1c** by microwave irradiation of thiazine **2c**.

Additionally, the fragmentation to pyridine **9** could be avoided by introducing an electron-withdrawing substituent in the product next to the N–C single bond, making it less prone to oxidation (Scheme 11). Indeed, when we used 4-(4-methoxyphenyl)-6-(2-(trifluoromethyl)phenyl)-3,6-dihydro-2*H*-1,3-thiazine-2-thione (**2c**) as the reaction input under the optimized reaction conditions we obtained bicyclic aminal **1c** in a rewarding 86% yield.

**Scheme 12:**
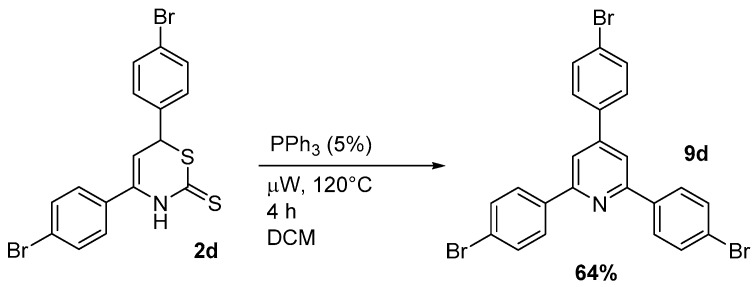
Synthesis of **1c** by microwave irradiation of thiazine **2c**.

However, when 4,6-bis(4-bromophenyl)-3,6-dihydro-2*H*-1,3-thiazine-2-thione was employed, 64% of pyridine **9d** was isolated (Scheme 12). The fragmentation towards the pyridines is apparently favored even with bromine substituents.

Finally, in order to investigate the potential role of the serendipitously added triphenylphosphine in this reaction a ^31^P-NMR spectrum was taken of the crude reaction mixture of the reaction towards **1d**. This spectrum shows the characteristic signals of both triphenylposhine oxide (27.0 ppm) and tripenylphosphine suflide (42.5 ppm), suggesting the triphenylphosphine acts as a scavenger for reactive oxygen and sulfur species in this reaction and thereby giving a cleaner reaction outcome [23].

## 3. Experimental Section

The reactions were carried out under inert atmosphere in a Biotage Initiator^+^ dedicated microwave reactor (Biotage, Uppsala, Sweden). ^1^H and ^13^C nuclear magnetic resonance (NMR) spectra were recorded on a Bruker Avance 500 (500.23 MHz and 125.78 MHz respectively, Bruker, Billerica, MA, USA) with chemical shifts (δ) reported in ppm downfield from tetramethylsilane. Electrospray Ionization (ESI) mass spectrometry was carried out using a Bruker micrOTOF-Q instrument in positive ion mode (capillary potential of 4500 V, Bruker, Billerica, MA, USA). Column chromatography was performed with flash silica gel (40–63 µm) and a mixture of ethyl acetate and cyclohexane. Compounds on Thin Layer Chromatography (TLC) were visualized by UV detection (CAMAG, Muttenz, Switserland). DCM was dried and distilled from sodium hydride prior to use. All commercially available reagents were used without further purification. The protocol for the in situ formation of 1-azadiene **4** can be found in our earlier report on thiazine-2-thiones [12]. X-ray diffraction date were collected on an Agilent SuperNova instrument (Agilent, Santa Clara, CA, USA) with Cu Ka radiation and focusing optics at 100 K, in w-scans.

CCDC 1415230 contains the supplementary crystallographic data for this paper. These data can be obtained free of charge via www.ccdc.cam.ac.uk/conts/retrieving.html (or from the CCDC, 12 Union Road, Cambridge CB2 1EZ, UK; fax: +44-1223-336-033; e-mail: deposit@ccdc.cam.ac.uk)


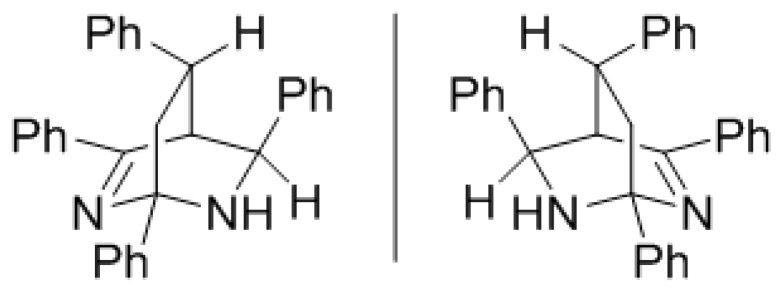


*(1S,4S,5S,8S)/(1R,4R,5R,8R)-1,3,5,8-Tetraphenyl-2,6-diazabicyclo[2.2.2]oct-2-ene* (**1a**): 4,6-diphenyl-3,6-dihydro-2*H*-1,3-thiazine-2-thione (425 mg, 1.50 mmol, 1 eq) was dissolved in dry dichloromethane (15.0 mL, 0.1 M) together with triphenylphosphine (20 mg, 5 mol %) in a flame dried microwave tube. The then capped tube was irradiated at 120 °C for 4 h using a dedicated microwave reactor. Concentration *in vacuo* and column chromatography afforded **1a** as a colorless solid (131 mg, 0.32 mmol, 42%). X-ray quality crystals were obtained by forced evaporation of an acetonitrile solution under a gentle stream of nitrogen. ^1^H-NMR (CDCl_3_) δ (ppm) 8.24 (d, *J* = 7.5 Hz, 2H), 7.79 (d, *J* = 7.5 Hz, 2H), 7.67 (d, *J* = 7.5 Hz, 2H), 7.51 (d, *J* = 7.5 Hz, 2H), 7.48 (d, *J* = 7.5 Hz, 2H), 7.43–7.33 (m, 5H), 7.10–7.05 (m, 3H), 6.89–6.84 (m, 2H), 4.16 (s, 1H), 3.62 (s, 1H), 3.26 (m, 1H), 2.74 (dd, *J* = 10 Hz, *J* = 13.5 Hz, 1H), 1.97 (bs, NH), 1.86 (dd, *J* = 6 Hz, *J* = 13.5 Hz, 1H); ^13^C-NMR (CDCl_3_) δ (ppm) 173.87 (C), 145.46 (C), 144.11 (C), 142.41 (C), 137.57 (C), 130.26 (CH), 128.53 (CH), 128.39 (CH), 128.34 (CH), 128.25 (CH), 127.55 (CH), 127.44 (CH), 127.13 (CH), 126.93 (CH), 126.61 (CH,) 126.34 (CH), 75.14 (C), 57.80 (CH), 47.48 (CH), 42.97 (CH_2_), 35.05 (CH); HRMS [M + H]^+^ 415.2191 (calc. C_30_H_27_N_2_^+^, 415.2169); Melting point (crushed crystals from acetonitrile) 180–183 °C; X-ray diffraction C_30_H_26_N_2_, *a* = 9.8194(6)Å, *b* = 10.4957(6)Å, *c* = 11.9141(6)Å, α = 76.408(5)°, β = 87.802(5)°, γ = 66.690(6)°, V = 1094.03(12)Å^3^, Z = 2, crystal size (mm) 0.50 × 0.45 × 0.15, No. of measured reflections 9136, 4492 independent, 4076 observed [*I* > 2σ(*I*)], *R*_int_ 0.034, *R*[*F*^2^ > 2σ(*F*^2^)] 0.052, *wR*(*F*^2^) 0.143, *GooF* 1.03. No. of parameters 292, Δρ_max_, Δρ_min_ (e Å^−3^) 0.34, −0.32.


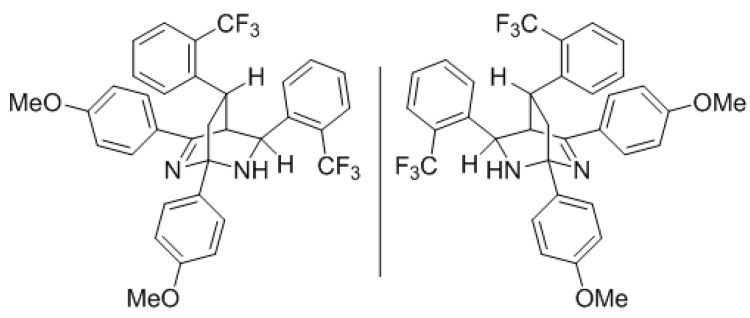


*(1R,4R,5R,8S)/(1R,4R,5R,8R)-1,3-bis(4-Methoxyphenyl)-5,8-bis(2-(trifluoromethyl)-phenyl)-2,6-diazabicyclo[2.2.2]oct-2-ene* (**1c**): 4-(4-methoxyphenyl)-6-(2-(trifluoromethyl)phenyl)-3,6-dihydro-2*H*-1,3-thiazine-2-thione (191 mg, 0.50 mmol, 1 eq) was dissolved in dry dichloromethane (5 mL, 0.1 M) together with triphenylphosphine (4 mg, 5 mol %) in a flame dried microwave tube. The then capped tube was irradiated at 120 °C for 4 h using a dedicated microwave reactor. Concentration *in vacuo* and recrystallization from EtOH afforded 1c as an off-white solid (131 mg, 0.22 mmol, 86%). ^1^H-NMR (CDCl_3_) δ (ppm) 8.62 (d, *J* = 6 Hz, 1H), 8.14 (d, *J* = 6 Hz, 2H), 7.82–7.72 (m, 4H), 7.51 (t, *J* = 6 Hz, 1H), 7.45 (d, *J* = 5.5 Hz, 1H), 7.18–7.10 (m, 2H), 7.07 (d, *J* = 6.5 Hz, 1H), 7.04 (d, *J* = 7.5 Hz, 2H), 6.89 (d, *J* = 6.5 Hz, 2H), 4.55 (s, 1H), 3.89 (s, 3H), 3.85 (s, 3H), 3.60 (s, 1H), 2.88 (t, *J* = 9 Hz, 1H), 2.04 (s, 1H), 1.91–1.80 (m, 1H), 1.62 (bs, 1H); ^13^C-NMR (CDCl_3_) δ (ppm) 173.1 (C), 161.7 (C), 159.0 (C), 142.8 (C), 139.8 (C), 137.4 (C), 131.8 (CH), 131.7 (CH), 130.1 (CH), 129.3 (CH), 128.0 (CH), 128.4–127.2 (C, m), 127.8 (CH, q, *J* = 6Hz), 127.5 (CH), 126.2 (CH), 125.9 (C), 125.5 (C, q, *J* = 6Hz), 125.1 (C), 113.8 (CH), 113.6 (CH), 74.4 (C), 55.3 (CH_3_), 55.3 (CH_3_), 54.8 (CH), 44.2 (CH_2_), 43.7 (CH), 29.3 (CH); HRMS [M + H]^+^ 611.2151 (calc. C_34_H_29_F_6_N_2_O_2_^+^, 611.2128); Melting point 174–177 °C.


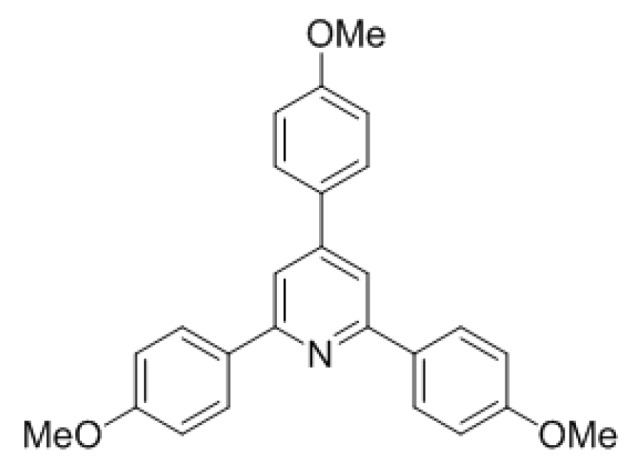


*2,4,6-tris(4-Methoxyphenyl)pyridine* (**9b**): 4,6-bis(4-methoxyphenyl)-3,6-dihydro-2*H*-1,3-thiazine-2-thione (154 mg, 0.45 mmol, 1 eq) was dissolved in dry dichloromethane (4.5 mL, 0.1 M) together with triphenylphosphine (4 mg, 5 mol %) in a flame dried microwave tube. The then capped tube was irradiated at 120 °C for 4 h using a dedicated microwave reactor. Concentration *in vacuo* and column chromatography afforded **9b** as a yellow oil (70 mg, 0.18 mmol, 79%). ^1^H-NMR (CDCl_3_) δ (ppm) 8.15 (d, *J* = 7 Hz, 4H), 7.75 (s, 2H), 7.69 (d, *J* = 7 Hz, 2H), 7.04 (m, 6H), 3.89 (s, 9H); ^13^C-NMR (CDCl_3_) δ (ppm) 160.4 (C), 160.4 (C), 149.5 (C), 132.4 (C), 131.6 (C), 128.4 (CH), 128.3 (CH), 115.3 (CH), 114.5 (CH), 114.0 (CH), 55.5 (CH_3_), 55.4 (CH_3_); HRMS [M + H]^+^ 198.1759 (calc. C_26_H_24_NO_3_^+^, 398.1751); Spectra are in accordance with alternative route [24].


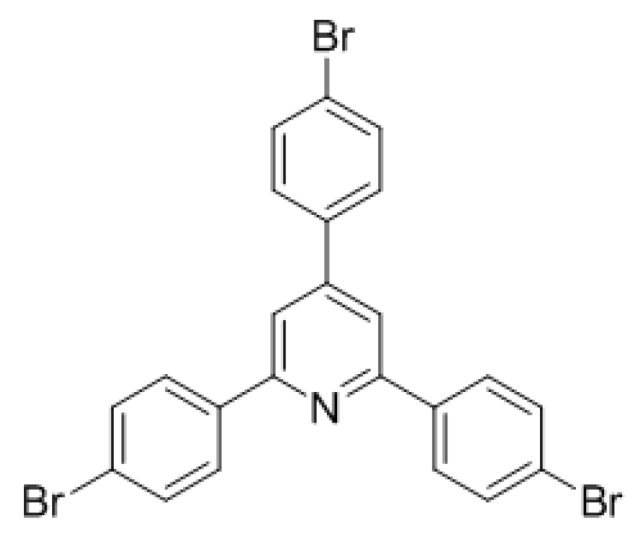


*2,4,6-tris(4-Bromophenyl)pyridine* (**9d**): 4,6-bis(4-bromophenyl)-3,6-dihydro-2*H*-1,3-thiazine-2-thione (132 mg, 0.30 mmol, 1 eq) was dissolved in dry dichloromethane (3.0 mL, 0.1 M) together with triphenylphosphine (4 mg, 5 mol %) in a flame dried microwave tube. The then capped tube was irradiated at 120 °C for 4 h using a dedicated microwave reactor. Concentration *in vacuo* recrystallization from EtOH/MeOH afforded 9d as a beige solid (52 mg, 0.10 mmol, 64%). ^1^H-NMR (CDCl_3_) δ (ppm) 8.05 (d, *J* = 6.5 Hz, 4H), 7.82 (s, 2H), 7.68–7.58 (m, 8H); ^13^C-NMR (CDCl_3_) δ (ppm) 156.7 (C), 149.4 (C), 138.0 (C), 137.6 (C), 132.4 (CH), 131.9 (CH), 128.7 (CH), 128.7 (CH), 123.8 (C), 123.7 (C), 116.8 (CH) ; HRMS [M + H]^+^ 541.8753 (calc. C_23_H_13_Br_3_N^+^, 541.8749); Melting point 237 °C (decomp.).

## 4. Conclusions

We reported a new route towards the 1-azadiene dimers, 1,3,5,8-tetraaryl-2,6-diazabicyclo[2.2.2]oct-2-enes. The products were formed diastereoselectively favoring the all-*S*/all-*R* racemate. The structure was confirmed by detailed NMR studies and X-ray crystallography. These bicyclic product are not well known, based on the fact only few reports on them were published, and could therefore represent an undiscovered part of chemical space with potential biological activity.

We envisioned a *retro*-hetero-Diels-Alder reaction or ring opening of the initially formed thiazine-2-thione, liberating the 1-azadiene and/or its CS_2_ adduct and subsequent *aza*-Diels-Alder dimerization, followed by a second ring closure to account for product formation. The thiazine-2-thione serves as a “masked” 1-azadiene and could prove a convenient synthetic substitute for the use of otherwise quite reactive intermediates.

However, when mesomerically electron-rich thiazines were employed, the formation of triaryl pyridines was observed. We think these were formed via a formal *retro*-hetero-Diels-Alder from the intermediate 1,3,5,8-tetra-aryl-2,6-diazabicyclo-[2.2.2]oct-2-enes after initial oxidation. This hypothesis was supported by the observation that reaction of a dihydrothiazine thione with an electron-deficient aryl substituent (which is less likely to undergo this oxidation) did not afford the triarylpyridine, but exclusively the azadiene dimer in very good yield.

## Supplementary Materials

Supplementary materials are available online.
