# Supplementary material for: Thiazine-2-thiones as Masked 1-Azadienes in Cascade Dimerization Reactions
Source: Molecules. 2017 Mar 28;22(4):541. doi: 10.3390/molecules22040541 (PMC6154663; doi:10.3390/molecules22040541)
Supplement: Supplementary file 1 [file molecules-22-00541-s001.pdf]

AK340 - F2

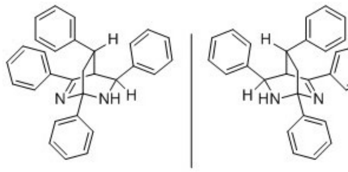

1a

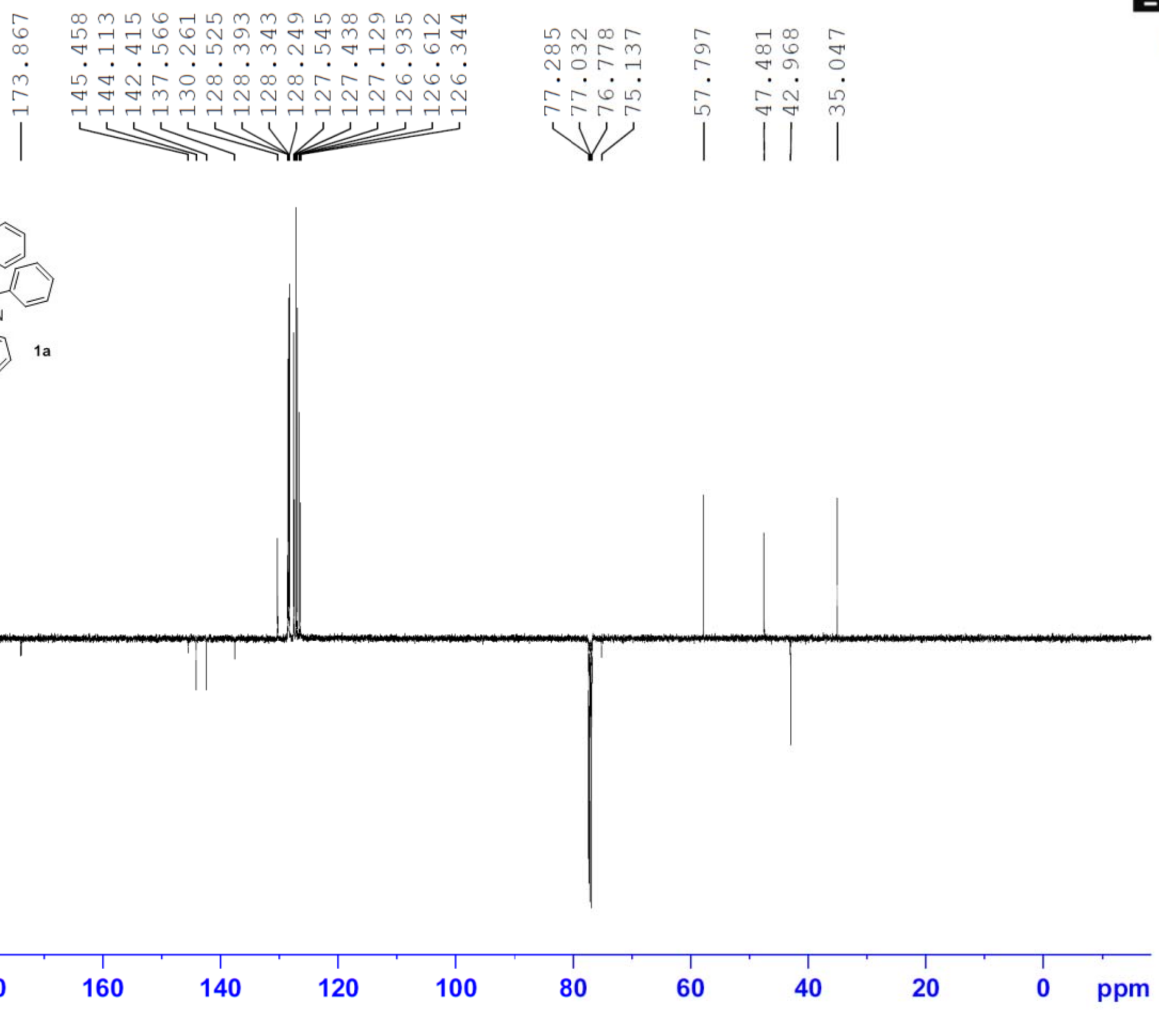


BRUKER

Current Data Parameters
NAME AK340
EXPNO 2
PROCNO 1

F2 - Acquisition Parameters
Date_ 20120913
Time 15.35
INSTRUM spect
PROBHD 5 mm CPTCI 1H-
PULPROG jmod
TD 65536
SOLVENT CDCl3
NS 192
DS 4
SWH 29761.904 Hz
FIDRES 0.454131 Hz
AQ 1.1010048 sec
RG 2050
DW 16.800 usec
DE 6.50 usec
TE 296.1 K
CNST2 145.0000000
CNST11 1.0000000
D1 2.00000000 sec
D20 0.00689655 sec
TD0 1

======== CHANNEL f1 ========
NUC1 13C
P1 11.20 usec
P2 22.40 usec
PL1 -2.00 dB
PL1W 88.77790070 W
SFO1 125.7955118 MHz

======== CHANNEL f2 ========
CPDPRG[2 waltz16
NUC2 1H
PCPD2 80.00 usec
PL2 4.00 dB
PL12 25.28 dB
PL2W 8.72000027 W
PL12W 0.06494062 W
SFO2 500.2320009 MHz

F2 - Processing parameters
SI 32768
SF 125.7829340 MHz
WDW EM
SSB 0
LB 1.00 Hz
GB 0
PC 1.40

AK340

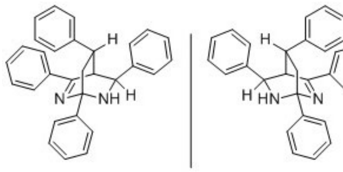

1a

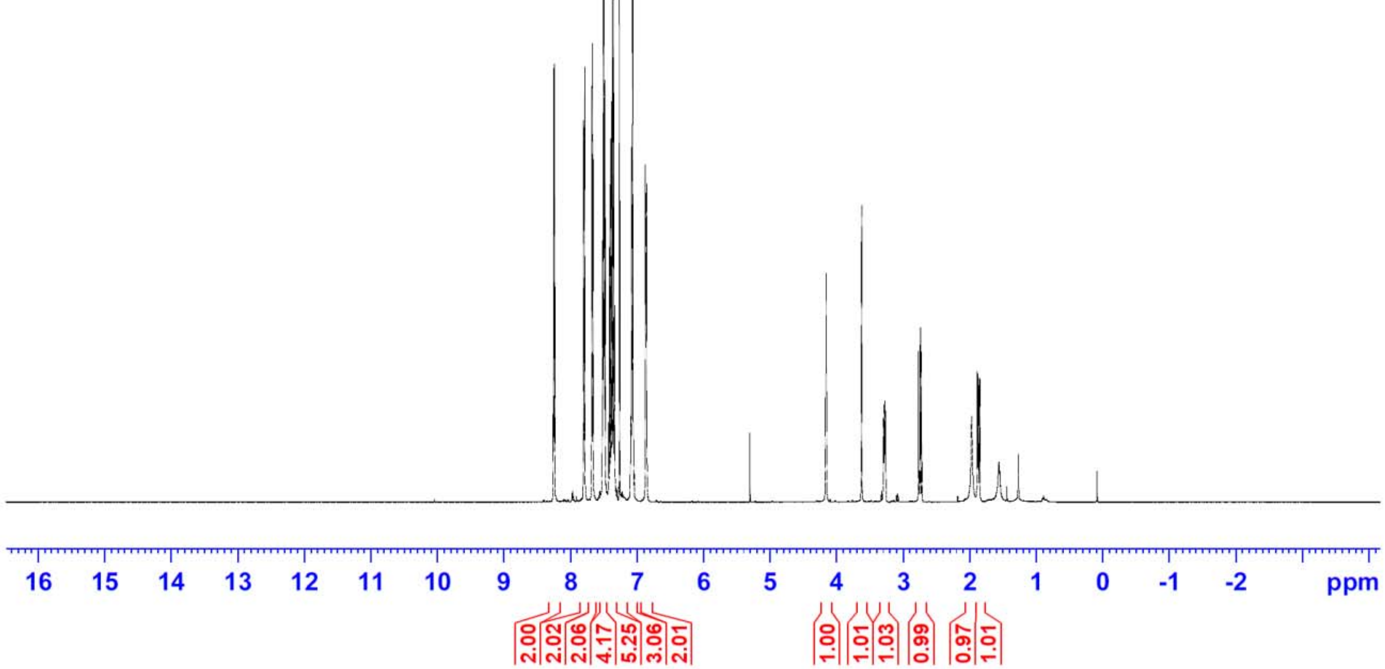


Current Data Parameters

| | |
|---|---|
| NAME | AK340 |
| EXPNO | 1 |
| PROCNO | 1 |

F2 - Acquisition Parameters

| | |
|---|---|
| Date_ | 20120913 |
| Time | 15.29 |
| INSTRUM | spect |
| PROBHD | 5 mm CPTCI 1H- |
| PULPROG | zg30 |
| TD | 65536 |
| SOLVENT | CDCl3 |
| NS | 16 |
| DS | 0 |
| SWH | 10330.578 Hz |
| FIDRES | 0.157632 Hz |
| AQ | 3.1719425 sec |
| RG | 14.2 |
| DW | 48.400 usec |
| DE | 6.50 usec |
| TE | 296.0 K |
| D1 | 2.00000000 sec |
| TD0 | 1 |

======== CHANNEL f1 ========

| | |
|---|---|
| NUC1 | 1H |
| P1 | 6.70 usec |
| PL1 | 4.00 dB |
| PL1W | 8.72000027 W |
| SFO1 | 500.2330891 MHz |

F2 - Processing parameters

| | |
|---|---|
| SI | 32768 |
| SF | 500.2300096 MHz |
| WDW | EM |
| SSB | 0 |
| LB | 0.30 Hz |
| GB | 0 |
| PC | 1.00 |

COSY
AK340

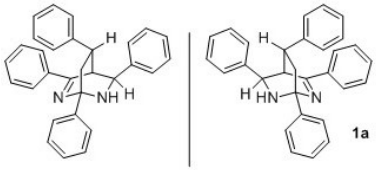

1a

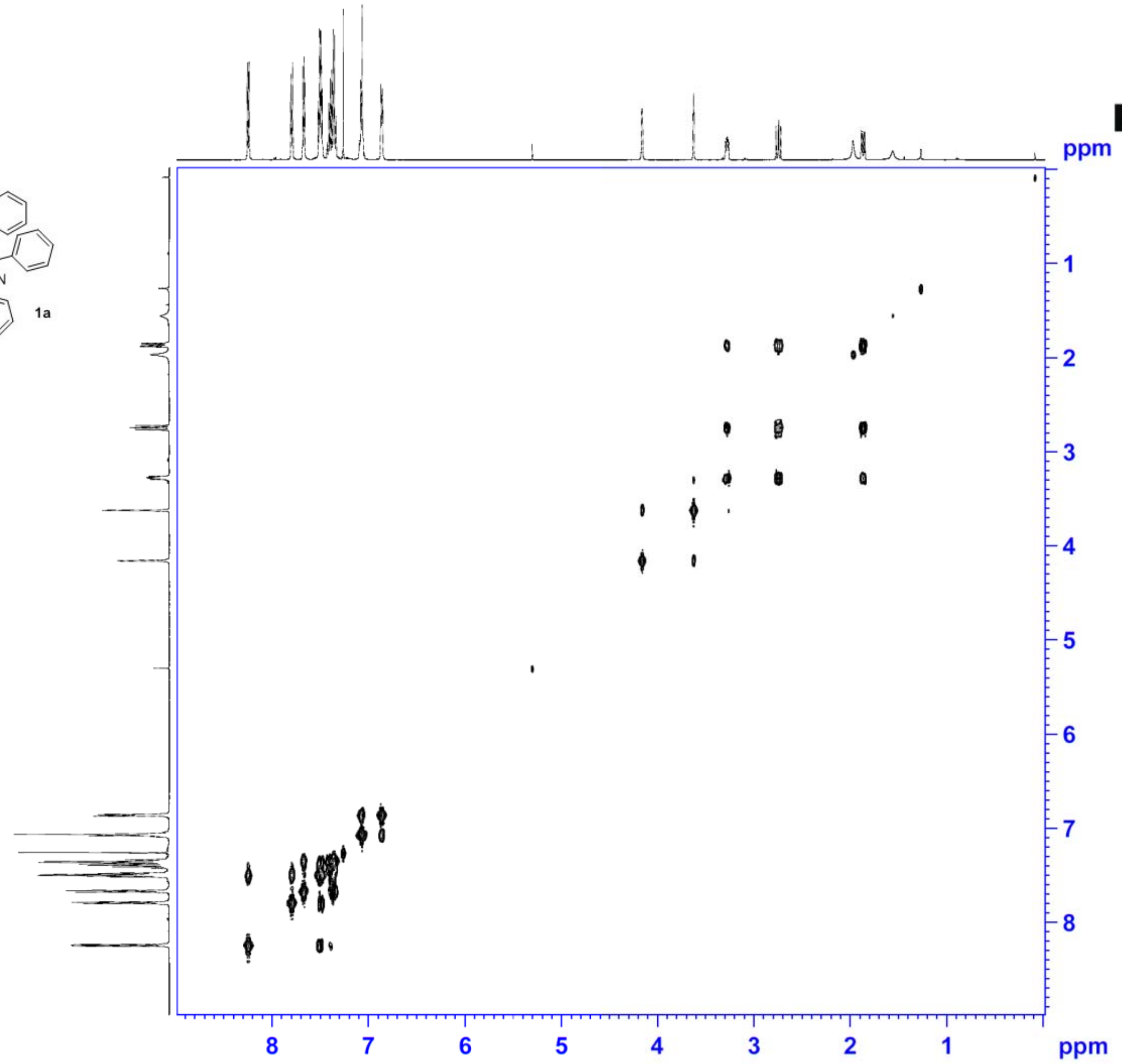


```
Current Data Parameters
NAME              AK340
EXPNO                 4
PROCNO                1

F2 - Acquisition Parameters
Date_          20120913
Time              16.00
INSTRUM           spect
PROBHD   5 mm CPTCI 1H-
PULPROG        cosygpqf
TD                 2048
SOLVENT           CDCl3
NS                    4
DS                   16
SWH            4504.504 Hz
FIDRES         2.199465 Hz
AQ            0.2273280 sec
RG                  322
DW              111.000 usec
DE                 6.50 usec
TE                296.1 K
D0           0.00000300 sec
D1           1.48689198 sec
D13          0.00000400 sec
D16          0.00020000 sec
IN0          0.00022210 sec

======== CHANNEL f1 ========
NUC1                 1H
P0                 6.70 usec
P1                 6.70 usec
PL1                4.00 dB
PL1W         8.72000027 W
SFO1        500.2322510 MHz

===== GRADIENT CHANNEL =====
GPNAM[1]       SINE.100
GPZ1              10.00 %
P16             1000.00 usec

F1 - Acquisition parameters
TD                  128
SFO1           500.2323 MHz
FIDRES        70.345161 Hz
SW                9.000 ppm
FnMODE               QF

F2 - Processing parameters
SI                 1024
SF          500.2300097 MHz
WDW                SINE
SSB        0
LB         0 Hz
GB         0
PC                 4.00

F1 - Processing parameters
SI                 1024
MC2                  QF
SF          500.2300084 MHz
WDW                SINE
SSB        0
LB         0 Hz
GB         0
```

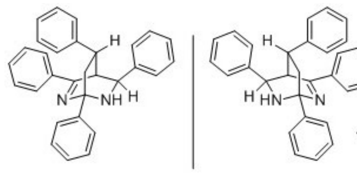

1a

HMBC
AK340

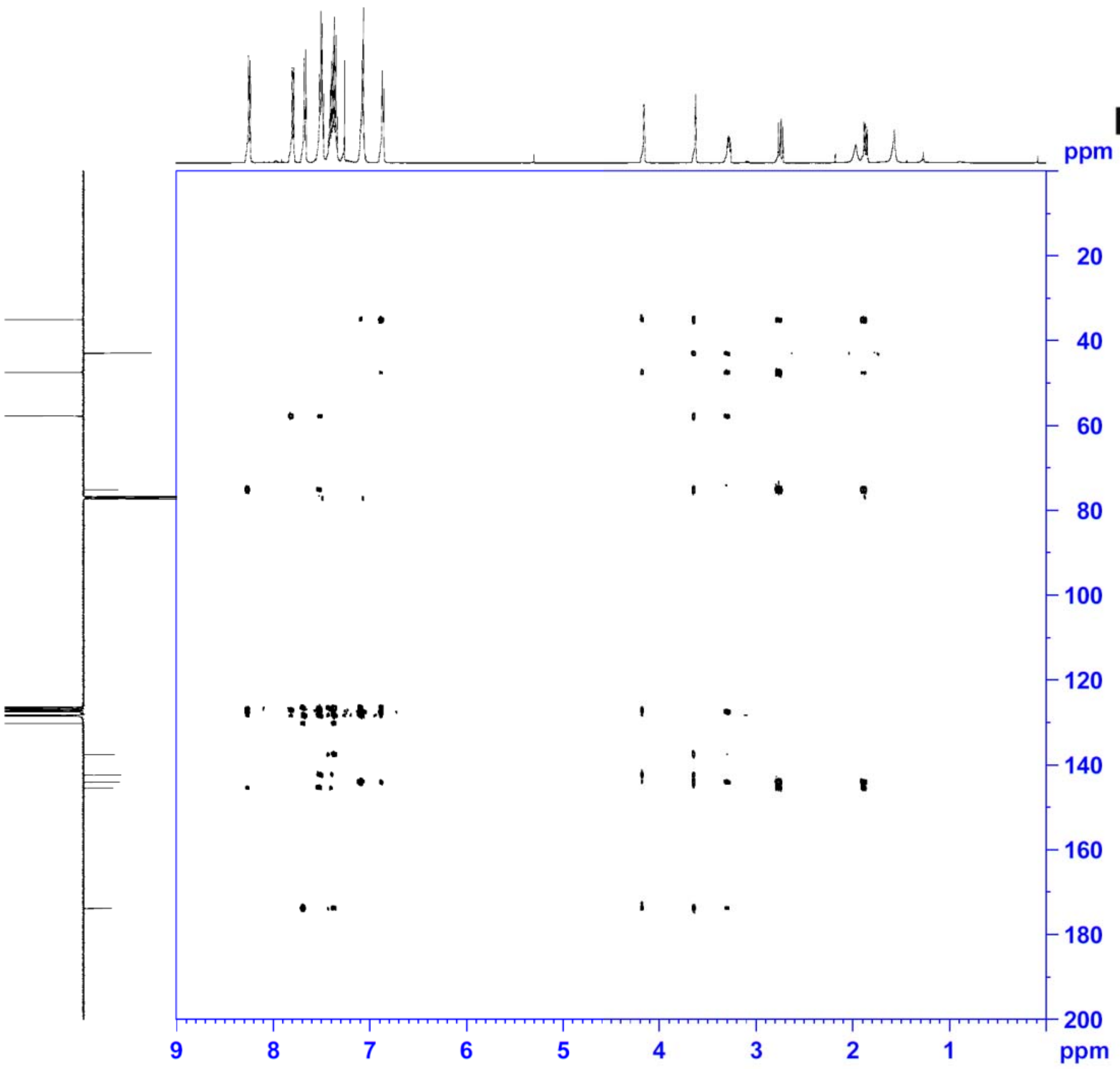


```
Current Data Parameters
NAME               AK340
EXPNO                  6
PROCNO                 1

F2 - Acquisition Parameters
Date_           20120913
Time               16.38
INSTRUM            spect
PROBHD   5 mm CPTCI 1H-
PULPROG     hmbcgplpndqf
TD                  4096
SOLVENT            CDCl3
NS                     2
DS                    16
SWH             4504.504 Hz
FIDRES          1.099733 Hz
AQ             0.4546560 sec
RG                  2050
DW               111.000 usec
DE                  6.50 usec
TE                 296.1 K
CNST2        145.0000000
CNST13         4.0000000
D0            0.00000300 sec
D1            1.00000000 sec
D2            0.00344828 sec
D6            0.12500000 sec
D16           0.00020000 sec
IN0           0.00001985 sec

======== CHANNEL f1 ========
NUC1                  1H
P1                  6.70 usec
P2                 13.40 usec
PL1                 4.00 dB
PL1W          8.72000027 W
SFO1         500.2322510 MHz

======== CHANNEL f2 ========
NUC2                 13C
P3                 11.20 usec
PL2                -2.00 dB
PL2W         88.77790070 W
SFO2         125.7955123 MHz

====== GRADIENT CHANNEL =====
GPNAM[1]        SINE.100
GPNAM[2]        SINE.100
GPNAM[3]        SINE.100
GPZ1               50.00 %
GPZ2               30.00 %
GPZ3               40.10 %
P16              1000.00 usec

F1 - Acquisition parameters
TD                   256
SFO1            125.7955 MHz
FIDRES        196.555481 Hz
SW               200.000 ppm
FnMODE                QF

F2 - Processing parameters
SI                  4096
SF           500.2299992 MHz
WDW                 SINE
SSB      0
LB       0 Hz
GB       0
PC                  4.00

F1 - Processing parameters
SI                  2048
MC2                   QF
SF           125.7829340 MHz
WDW                 SINE
SSB      0
LB       0 Hz
GB       0
```

HMBC 15N
AK340

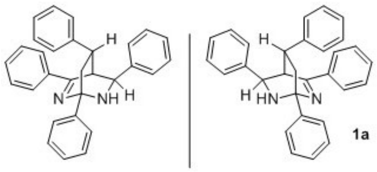

1a

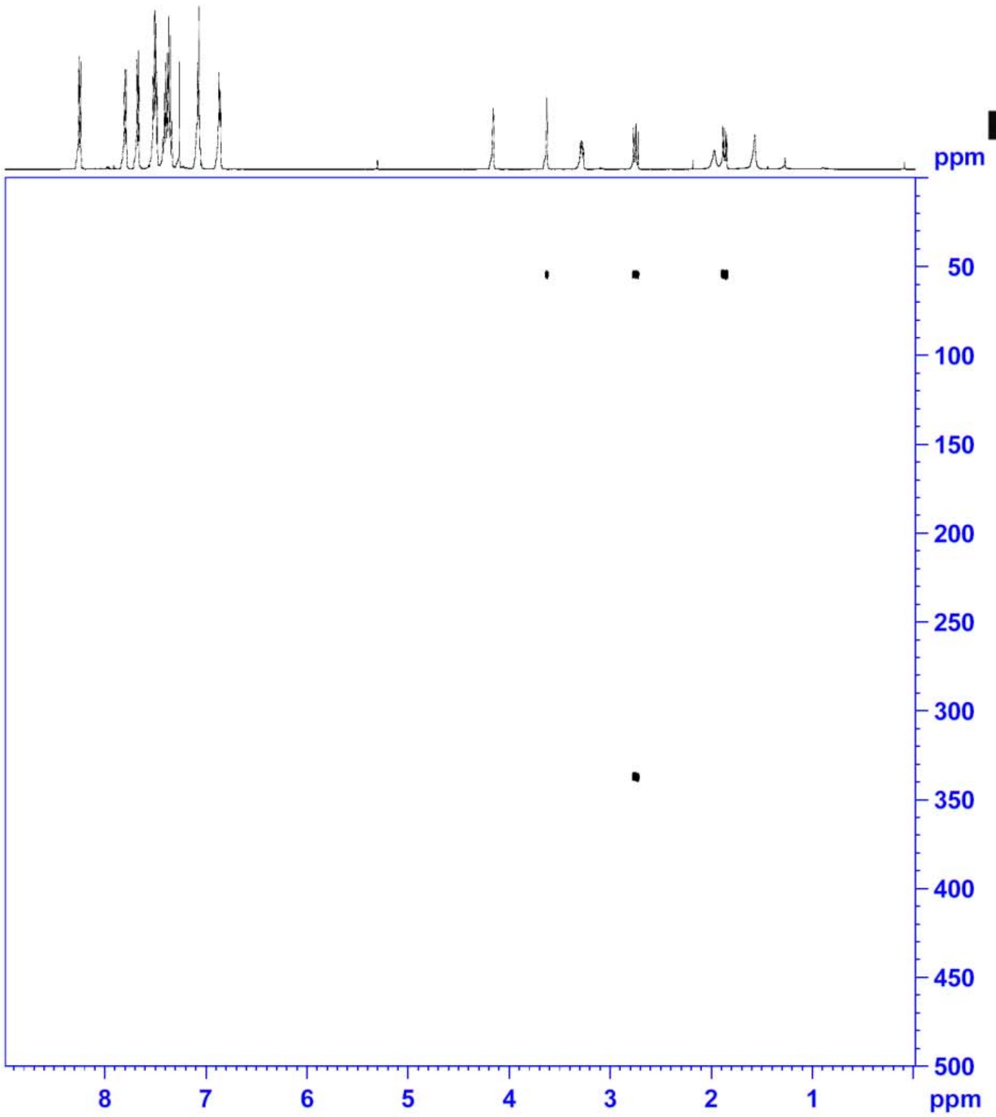


```
Current Data Parameters
NAME              AK340
EXPNO                 7
PROCNO                1

F2 - Acquisition Parameters
Date_          20120913
Time              16.54
INSTRUM           spect
PROBHD   5 mm CPTCI 1H-
PULPROG    hmbcf3gpndqf
TD                 4096
SOLVENT           CDCl3
NS                    4
DS                   16
SWH            4504.504 Hz
FIDRES         1.099733 Hz
AQ            0.4546560 sec
RG                 2050
DW              111.000 usec
DE                 6.50 usec
TE                296.0 K
CNST13        5.0000000
D0           0.00000300 sec
D1           1.25000000 sec
D6           0.10000000 sec
D16          0.00020000 sec
IN0          0.00001970 sec

======== CHANNEL f1 ========
NUC1                 1H
P1                 6.70 usec
P2                13.40 usec
PL1                4.00 dB
PL1W         8.72000027 W
SFO1        500.2322510 MHz

======== CHANNEL f3 ========
NUC3                15N
P21               30.00 usec
PL3               -1.70 dB
PL3W        85.08156586 W
SFO3         50.7005380 MHz

====== GRADIENT CHANNEL =====
GPNAM[1]       SINE.100
GPNAM[2]       SINE.100
GPNAM[3]       SINE.100
GPZ1              70.00 %
GPZ2              30.00 %
GPZ3              50.10 %
P16             1000.00 usec

F1 - Acquisition parameters
TD                  128
SFO1           50.70054 MHz
FIDRES       396.097961 Hz
SW              500.000 ppm
FnMODE               QF

F2 - Processing parameters
SI                 2048
SF          500.2300092 MHz
WDW                SINE
SSB                   2
LB         0 Hz
GB         0
PC                 4.00

F1 - Processing parameters
SI                 2048
MC2                  QF
SF           50.6878660 MHz
WDW                SINE
SSB        0
LB         0 Hz
GB         0
```

HSQC
AK340

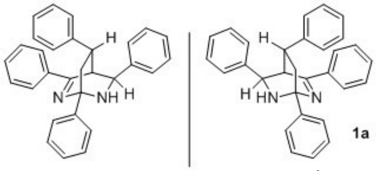

1a

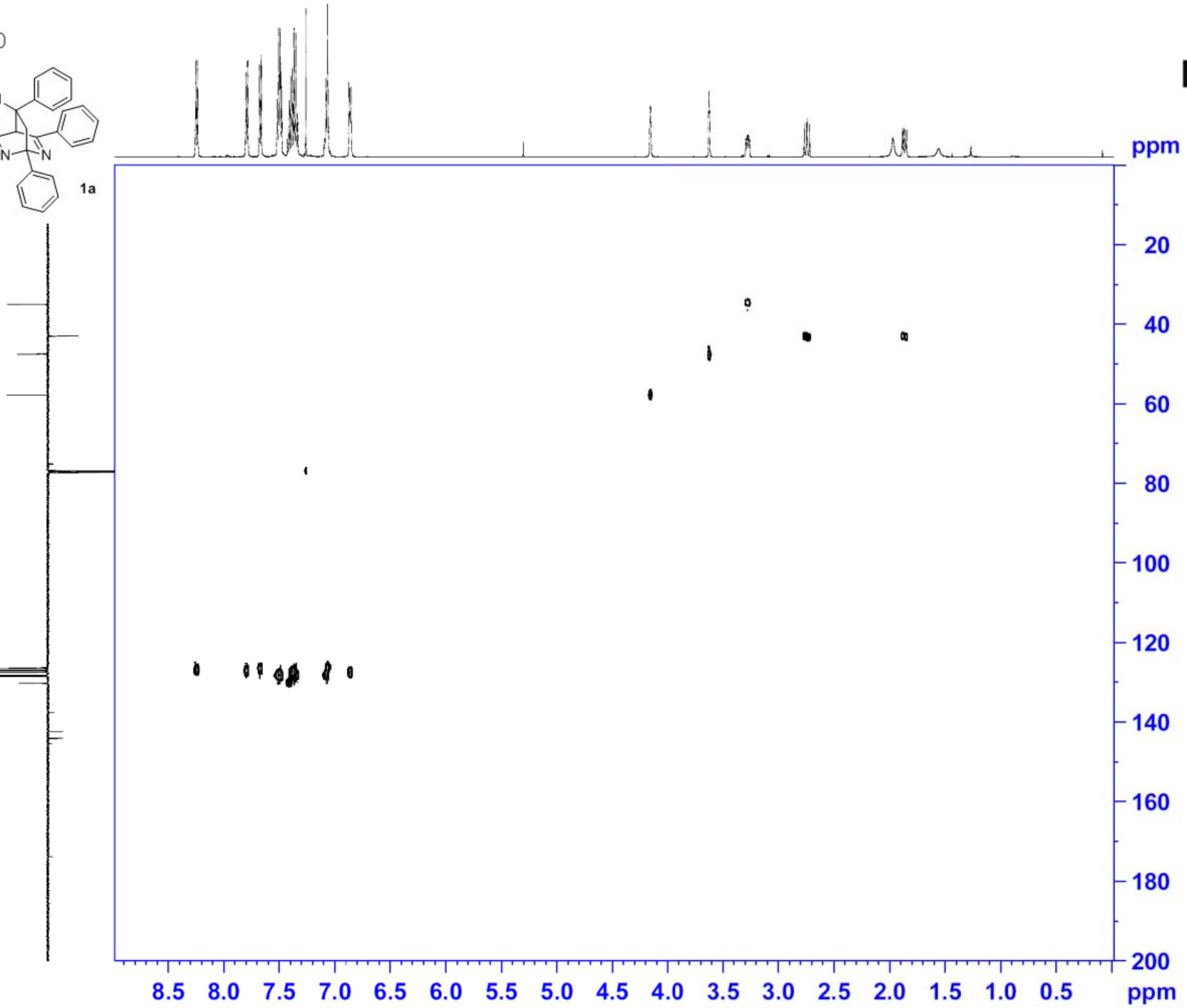


```
Current Data Parameters
NAME              AK340
EXPNO                 3
PROCNO                1

F2 - Acquisition Parameters
Date_          20120913
Time              15.44
INSTRUM           spect
PROBHD   5 mm CPTCI 1H-
PULPROG        hsqcetgp
TD                 2048
SOLVENT           CDCl3
NS                    4
DS                   16
SWH            4504.504 Hz
FIDRES         2.199465 Hz
AQ            0.2273280 sec
RG                 2050
DW              111.000 usec
DE                 6.50 usec
TE                296.0 K
CNST2       145.0000000
D0           0.00000300 sec
D1           1.50000000 sec
D4           0.00172414 sec
D11          0.03000000 sec
D13          0.00000400 sec
D16          0.00020000 sec
IN0          0.00001985 sec
ZGOPTNS

======== CHANNEL f1 ========
NUC1                 1H
P1                 6.70 usec
P2                13.40 usec
P28             1000.00 usec
PL1                4.00 dB
PL1W         8.72000027 W
SFO1        500.2322510 MHz

======== CHANNEL f2 ========
CPDPRG[2           garp
NUC2                13C
P3                11.20 usec
P4                22.40 usec
PCPD2             70.00 usec
PL2               -2.00 dB
PL12              13.92 dB
PL2W        88.77790070 W
PL12W        2.27145886 W
SFO2        125.7955123 MHz

====== GRADIENT CHANNEL =====
GPNAM[1]       SINE.100
GPNAM[2]       SINE.100
GPZ1              80.00 %
GPZ2              20.10 %
P16             1000.00 usec

F1 - Acquisition parameters
TD                  128
SFO1           125.7955 MHz
FIDRES       393.110962 Hz
SW              200.000 ppm
FnMODE    Echo-Antiecho

F2 - Processing parameters
SI                 2048
SF          500.2300095 MHz
WDW               QSINE
SSB                   2
LB        0 Hz
GB        0
PC                 4.00

F1 - Processing parameters
SI                 2048
MC2       echo-antiecho
SF          125.7829340 MHz
WDW               QSINE
SSB                   2
LB        0 Hz
GB        0
```

group OC

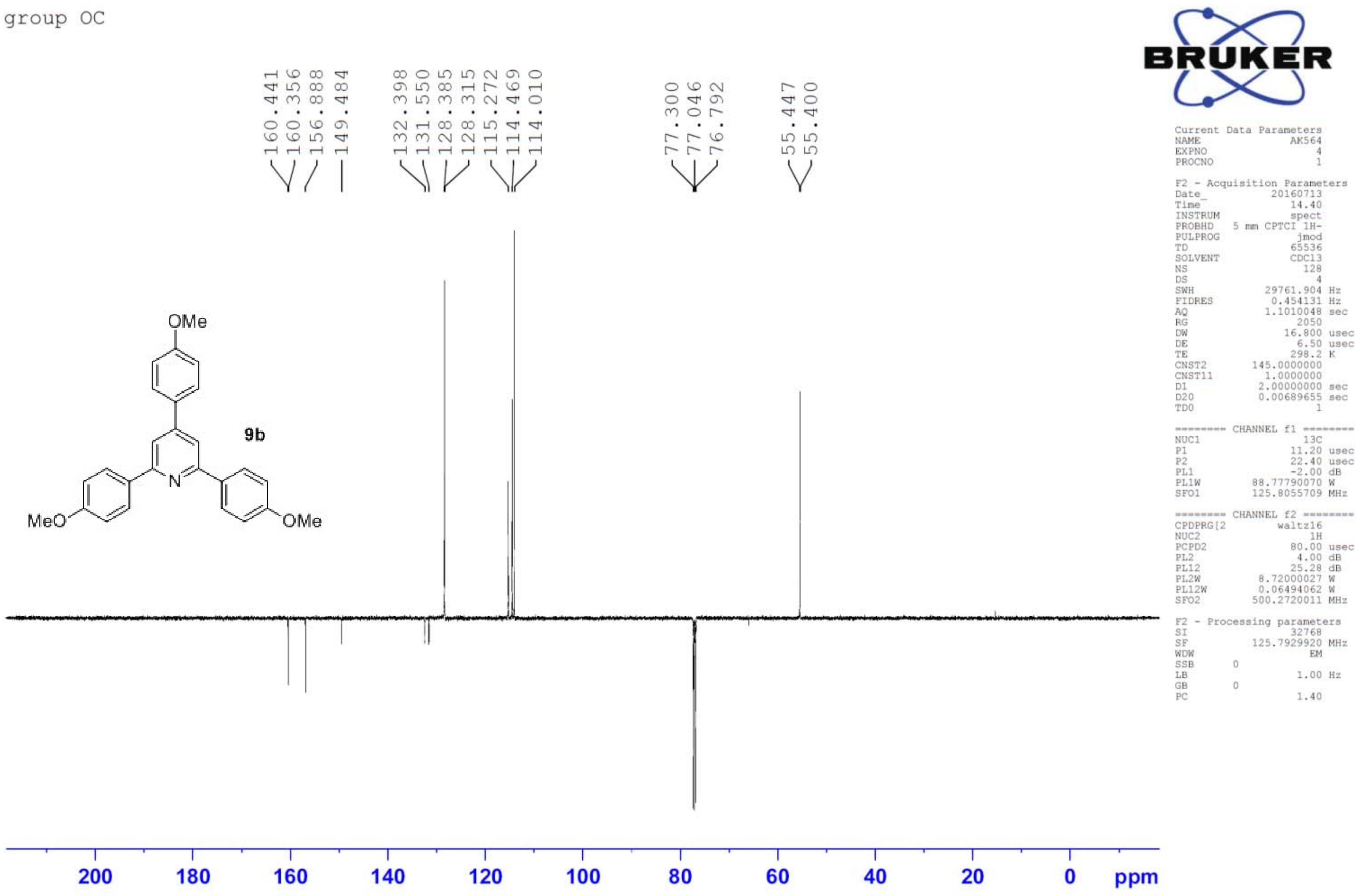
160.441
160.356
156.888
149.484
132.398
131.550
128.385
128.315
115.272
114.469
114.010
77.300
77.046
76.792
55.447
55.400
OMe
9b
MeO
N
OMe
200
180
160
140
120
100
80
60
40
20
0
ppm
BRUKER
Current Data Parameters
NAME AK564
EXPNO 4
PROCNO 1
F2 - Acquisition Parameters
Date_ 20160713
Time 14.40
INSTRUM spect
PROBHD 5 mm CPTCI 1H-
PULPROG jmod
TD 65536
SOLVENT CDCl3
NS 128
DS 4
SWH 29761.904 Hz
FIDRES 0.454131 Hz
AQ 1.1010048 sec
RG 2050
DW 16.800 usec
DE 6.50 usec
TE 298.2 K
CNST2 145.0000000
CNST11 1.0000000
D1 2.00000000 sec
D20 0.00689655 sec
TD0 1
======== CHANNEL f1 ========
NUC1 13C
P1 11.20 usec
P2 22.40 usec
PL1 -2.00 dB
PL1W 88.77790070 W
SFO1 125.8055709 MHz
======== CHANNEL f2 ========
CPDPRG[2 waltz16
NUC2 1H
PCPD2 80.00 usec
PL2 4.00 dB
PL12 25.28 dB
PL2W 8.72000027 W
PL12W 0.06494062 W
SFO2 500.2720011 MHz
F2 - Processing parameters
SI 32768
SF 125.7929920 MHz
WDW EM
SSB 0
LB 1.00 Hz
GB 0
PC 1.40

group OC

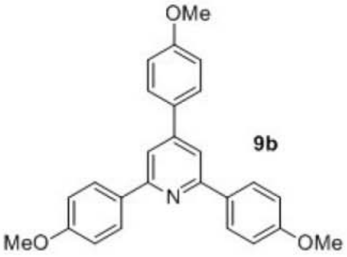


9b

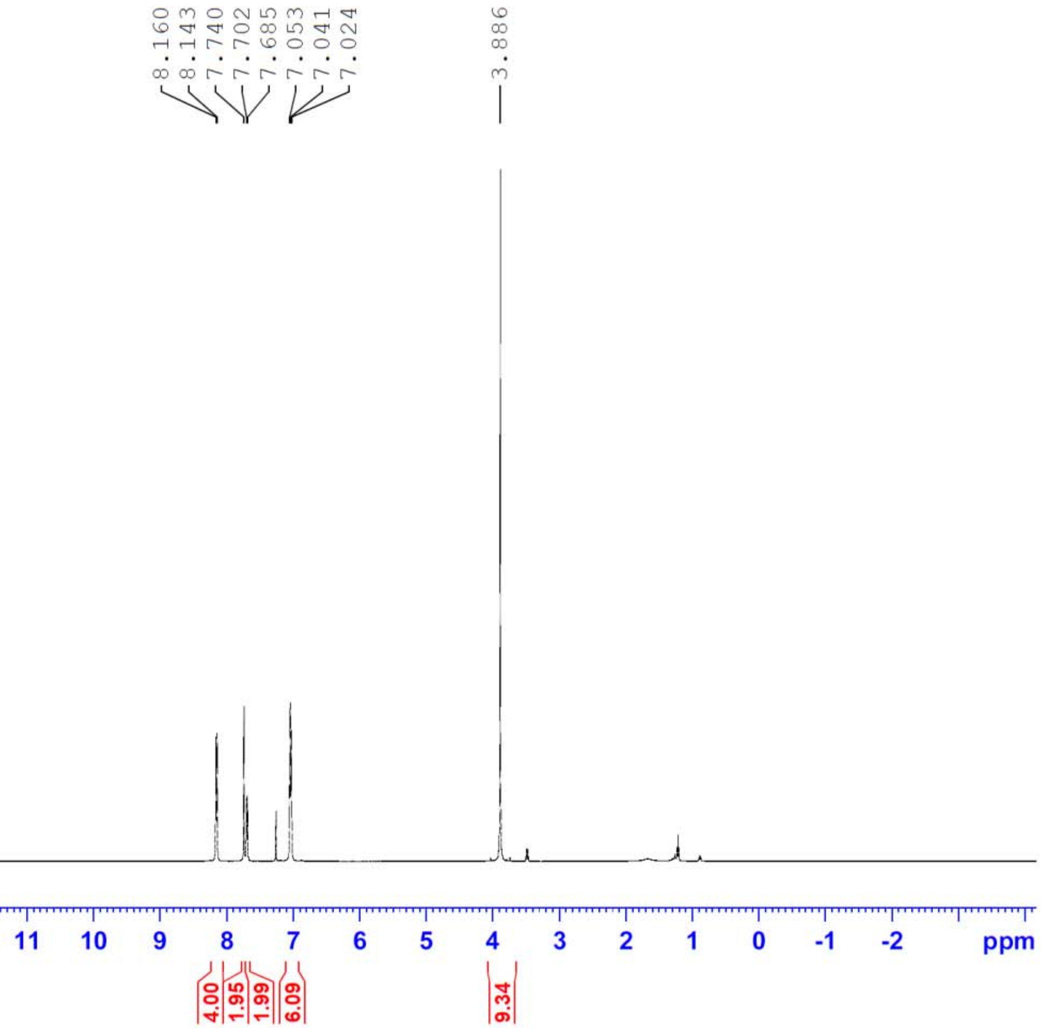


BRUKER

Current Data Parameters
NAME AK564
EXPNO 3
PROCNO 1

F2 - Acquisition Parameters
Date_ 20160713
Time 14.33
INSTRUM spect
PROBHD 5 mm CPTCI 1H-
PULPROG zg30
TD 65536
SOLVENT CDCl3
NS 16
DS 2
SWH 10330.578 Hz
FIDRES 0.157632 Hz
AQ 3.1719425 sec
RG 16
DW 48.400 usec
DE 6.50 usec
TE 298.3 K
D1 1.00000000 sec
TD0 1

======== CHANNEL f1 ========
NUC1 1H
P1 6.70 usec
PL1 4.00 dB
PL1W 8.72000027 W
SFO1 500.2730894 MHz

F2 - Processing parameters
SI 32768
SF 500.2700093 MHz
WDW EM
SSB 0
LB 0.30 Hz
GB 0
PC 1.00

group OC

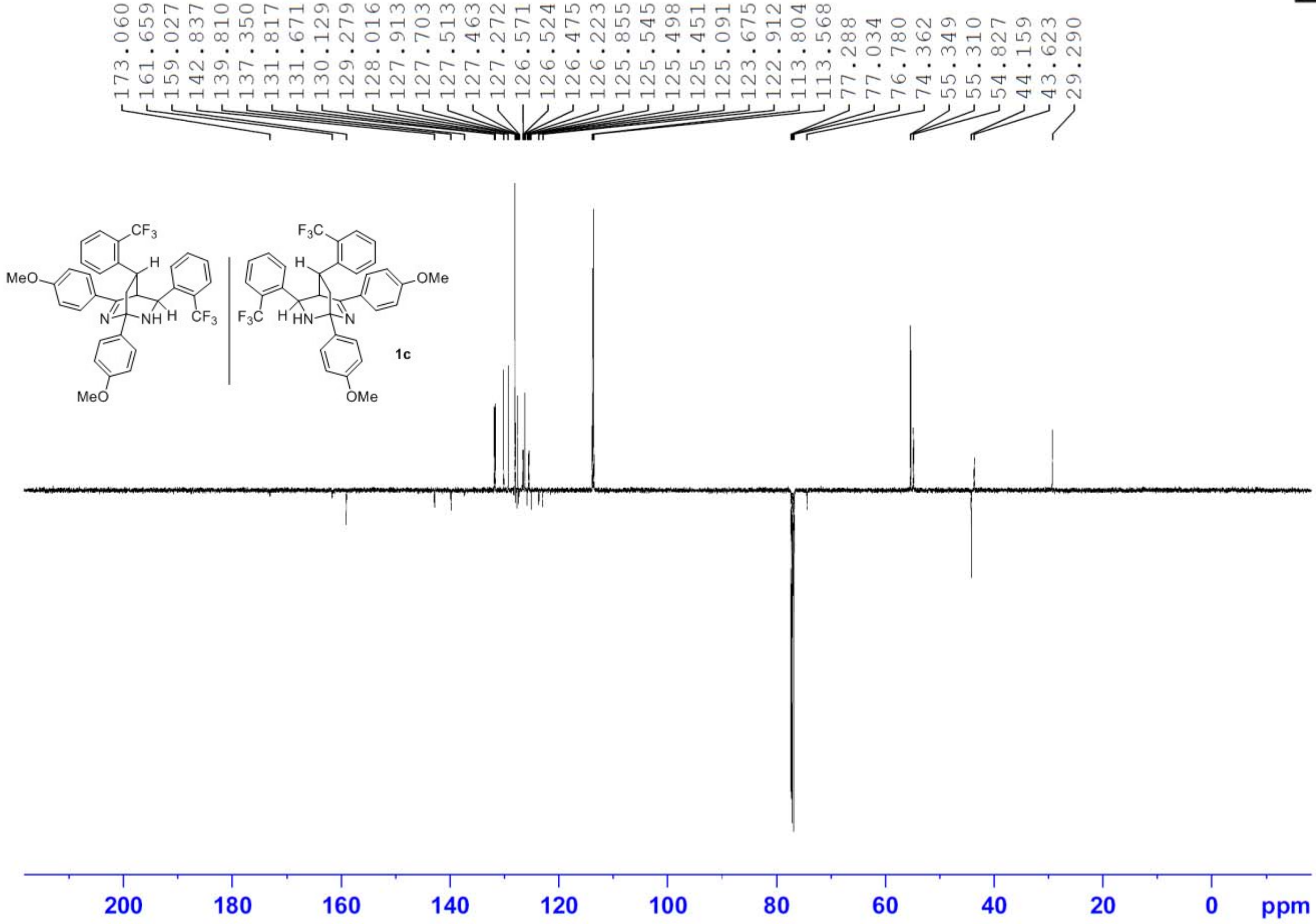


Current Data Parameters
NAME AK566
EXPNO 4
PROCNO 1

F2 - Acquisition Parameters
Date_ 20160719
Time 15.26
INSTRUM spect
PROBHD 5 mm CPTCI 1H-
PULPROG jmod
TD 65536
SOLVENT CDCl3
NS 256
DS 4
SWH 29761.904 Hz
FIDRES 0.454131 Hz
AQ 1.1010048 sec
RG 2050
DW 16.800 usec
DE 6.50 usec
TE 298.2 K
CNST2 145.0000000
CNST11 1.0000000
D1 2.00000000 sec
D20 0.00689655 sec
TD0 1

======== CHANNEL f1 ========
NUC1 13C
P1 11.20 usec
P2 22.40 usec
PL1 -2.00 dB
PL1W 88.77790070 W
SFO1 125.8055709 MHz

======== CHANNEL f2 ========
CPDPRG[2 waltz16
NUC2 1H
PCPD2 80.00 usec
PL2 4.00 dB
PL12 25.28 dB
PL2W 8.72000027 W
PL12W 0.06494062 W
SFO2 500.2720011 MHz

F2 - Processing parameters
SI 32768
SF 125.7929920 MHz
WDW EM
SSB 0
LB 1.00 Hz
GB 0
PC 1.40

group OC

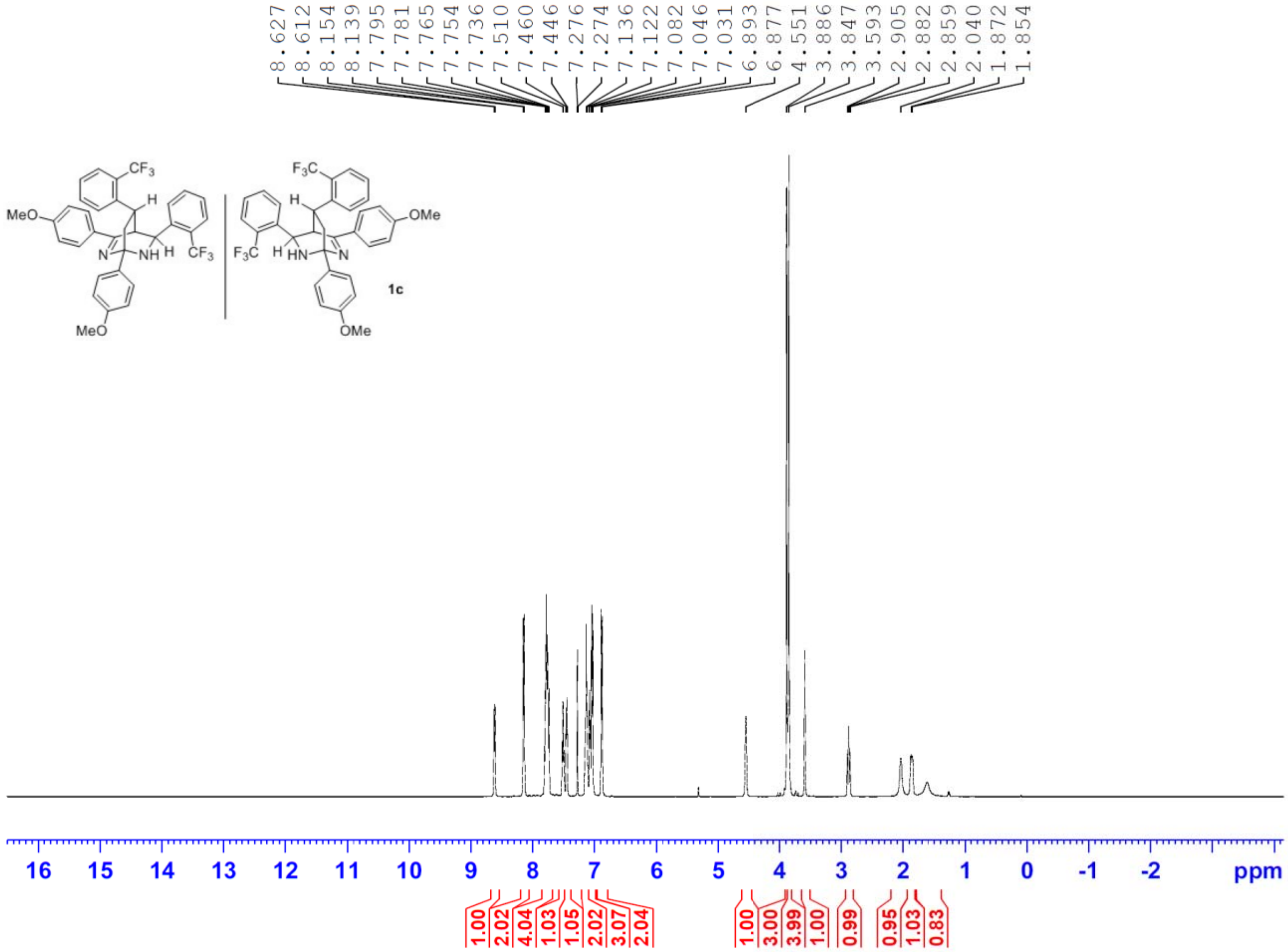


```
Current Data Parameters
NAME              AK566
EXPNO                 3
PROCNO                1

F2 - Acquisition Parameters
Date_          20160719
Time              15.12
INSTRUM           spect
PROBHD   5 mm CPTCI 1H-
PULPROG            zg30
TD                65536
SOLVENT           CDCl3
NS                   16
DS                    2
SWH          10330.578 Hz
FIDRES        0.157632 Hz
AQ           3.1719425 sec
RG                 14.2
DW               48.400 usec
DE                 6.50 usec
TE                298.2 K
D1         1.00000000 sec
TD0                   1

======== CHANNEL f1 ========
NUC1                 1H
P1                 6.70 usec
PL1                4.00 dB
PL1W         8.72000027 W
SFO1        500.2730894 MHz

F2 - Processing parameters
SI                32768
SF          500.2700000 MHz
WDW                  EM
SSB      0
LB                 0.30 Hz
GB       0
PC                 1.00
```

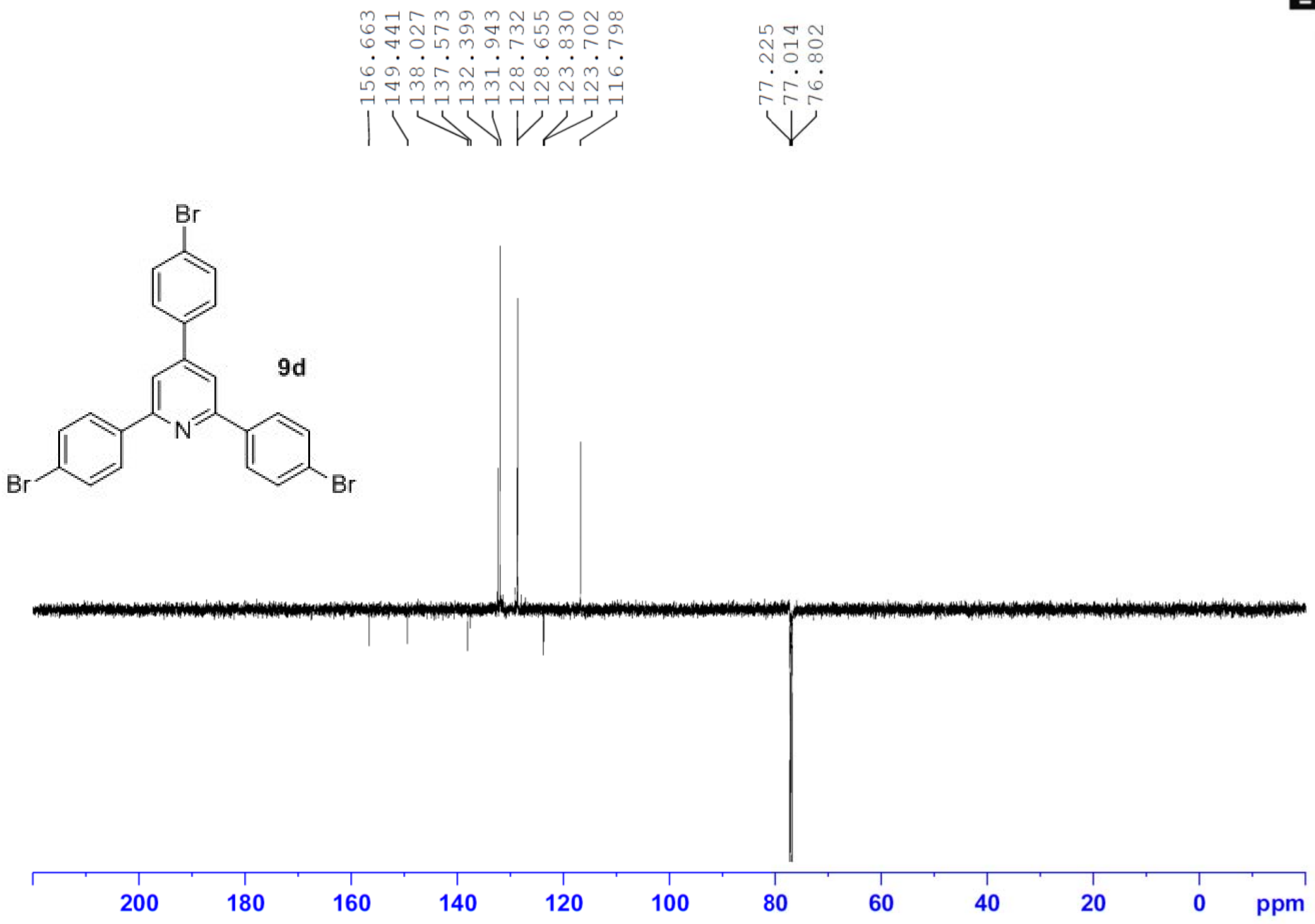


```
Current Data Parameters
NAME              AK572
EXPNO                 3
PROCNO                1

F2 - Acquisition Parameters
Date_          20170316
Time              13.51 h
INSTRUM           spect
PROBHD   Z117768_0067 (
PULPROG            jmod
TD                65536
SOLVENT           CDCl3
NS                   64
DS                    4
SWH           36231.883 Hz
FIDRES         0.552855 Hz
AQ            0.9043968 sec
RG               199.43
DW               13.800 usec
DE                18.00 usec
TE                298.0 K
CNST2       145.0000000
CNST11        1.0000000
D1           2.00000000 sec
D20          0.00689655 sec
TD0                   1
SFO1        150.9178988 MHz
NUC1                13C
P1                12.00 usec
P2                24.00 usec
PLW1        88.22599792 W
SFO2        600.1324005 MHz
NUC2                 1H
CPDPRG[2        waltz16
PCPD2             80.00 usec
PLW2         6.76100016 W
PLW12        0.06699187 W

F2 - Processing parameters
SI                32768
SF          150.9028085 MHz
WDW                  EM
SSB      0
LB                 1.00 Hz
GB       0
PC                 1.40
```

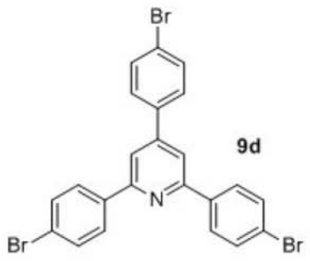


9d

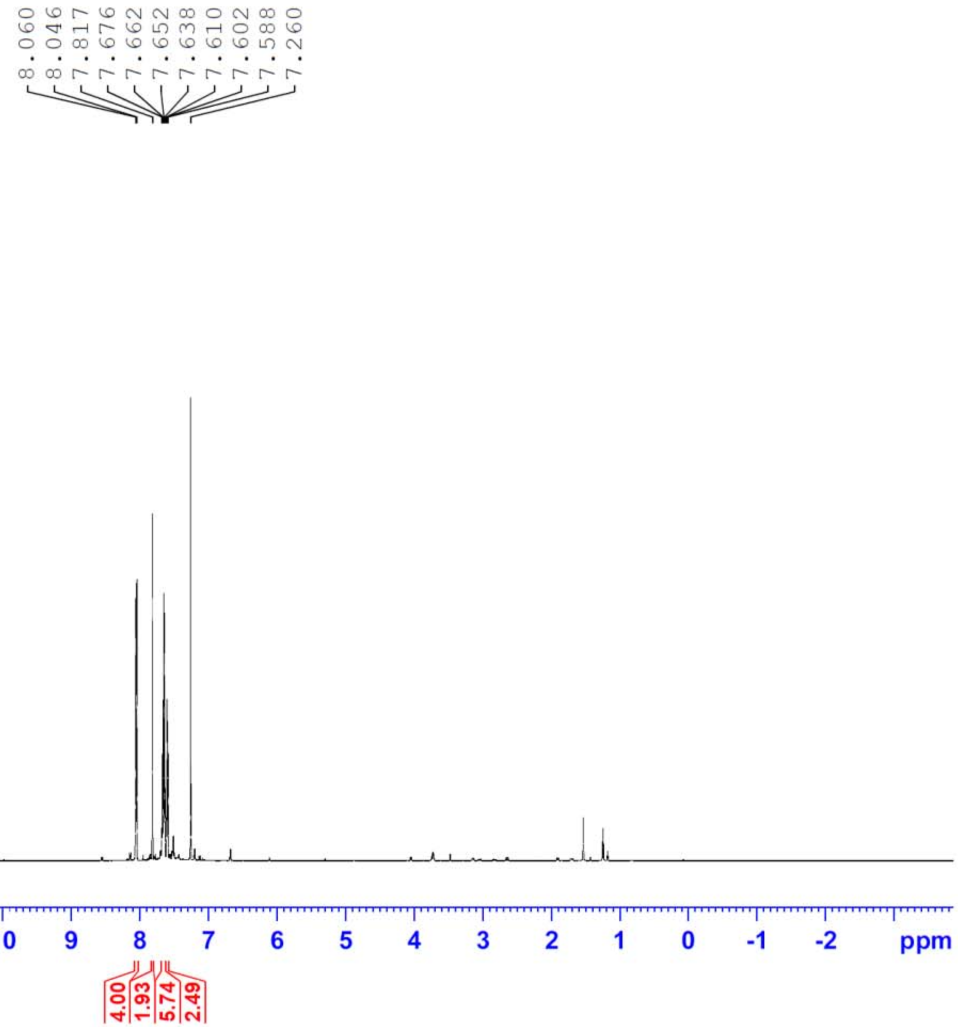


| Current Data Parameters | |
|---|---|
| NAME | AK572 |
| EXPNO | 2 |
| PROCNO | 1 |

| F2 - Acquisition Parameters | |
|---|---|
| Date_ | 20170316 |
| Time | 13.49 h |
| INSTRUM | spect |
| PROBHD | Z117768_0067 ( |
| PULPROG | zg30 |
| TD | 65536 |
| SOLVENT | CDCl3 |
| NS | 16 |
| DS | 2 |
| SWH | 12019.230 Hz |
| FIDRES | 0.183399 Hz |
| AQ | 2.7262976 sec |
| RG | 31.69 |
| DW | 41.600 usec |
| DE | 18.00 usec |
| TE | 298.0 K |
| D1 | 1.00000000 sec |
| TD0 | 1 |
| SFO1 | 600.1337058 MHz |
| NUC1 | 1H |
| P1 | 8.00 usec |
| PLW1 | 6.76100016 W |

| F2 - Processing parameters | |
|---|---|
| SI | 65536 |
| SF | 600.1300139 MHz |
| WDW | EM |
| SSB | 0 |
| LB | 0.30 Hz |
| GB | 0 |
| PC | 1.00 |

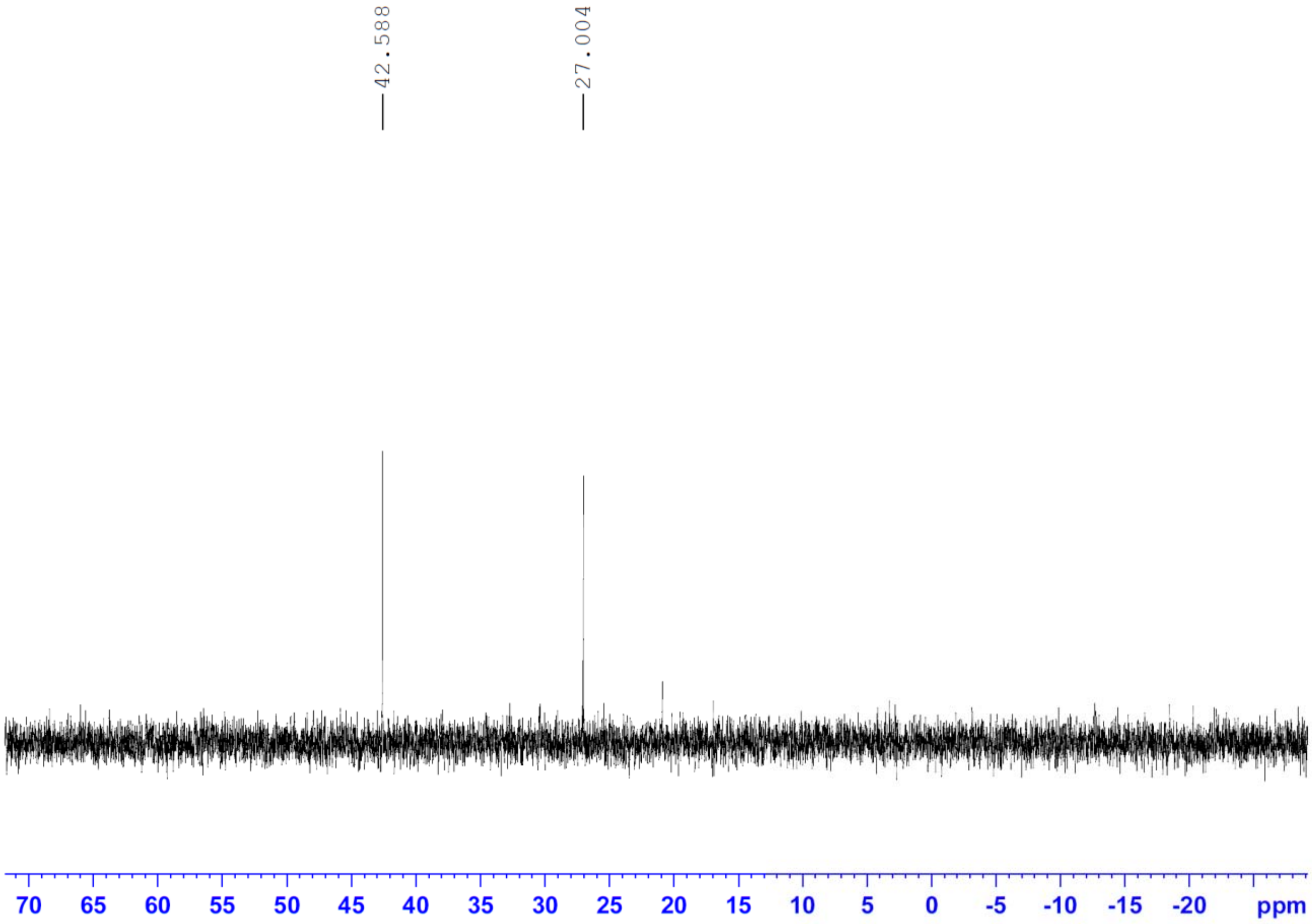


| | |
|---|---|
| Current Data Parameters | |
| NAME | AK572 |
| EXPNO | 1 |
| PROCNO | 1 |
| F2 - Acquisition Parameters | |
| Date_ | 20170316 |
| Time | 10.27 |
| INSTRUM | spect |
| PROBHD | 5 mm PABBO BB/ |
| PULPROG | zgpg30 |
| TD | 65536 |
| SOLVENT | None |
| NS | 64 |
| DS | 4 |
| SWH | 64935.066 Hz |
| FIDRES | 0.990830 Hz |
| AQ | 0.5046272 sec |
| RG | 22800 |
| DW | 7.700 usec |
| DE | 6.00 usec |
| TE | 293.0 K |
| D1 | 2.00000000 sec |
| d11 | 0.03000000 sec |
| DELTA | 1.89999998 sec |
| TD0 | 1 |
| ======== CHANNEL f1 ======== | |
| NUC1 | 31P |
| P1 | 6.25 usec |
| PL1 | -1.00 dB |
| SFO1 | 161.9674942 MHz |
| ======== CHANNEL f2 ======== | |
| CPDPRG[2 | waltz16 |
| NUC2 | 1H |
| PCPD2 | 80.00 usec |
| PL2 | 3.00 dB |
| PL12 | 18.00 dB |
| PL13 | 18.00 dB |
| SFO2 | 400.1316005 MHz |
| F2 - Processing parameters | |
| SI | 65536 |
| SF | 161.9755930 MHz |
| WDW | EM |
| SSB | 0 |
| LB | 1.00 Hz |
| GB | 0 |
| PC | 1.40 |
